# Characterization of human S-adenosyl-homocysteine hydrolase *in vitro* and identification of its potential inhibitors

**DOI:** 10.1080/14756366.2017.1370584

**Published:** 2017-09-21

**Authors:** Weiwei Hao, Yanhua Li, Qiuli Shan, Tian Han, Wencheng Li, Sheng He, Kongkai Zhu, Yumei Li, Xiaojun Tan, Jinsong Gu

**Affiliations:** School of Biological Science and Technology, University of Jinan, Jinan, China

**Keywords:** S-adenosyl-homocysteine hydrolase (SAHH), l-homocysteine (Hcy), *Pichia pastoris*, inhibitors, coniferyl alcohol

## Abstract

Human *S*-adenosyl-homocysteine hydrolase (SAHH, E.C.3.3.1.1) has been considered to be an attractive target for the design of medicines to treat human disease, because of its important role in regulating biological methylation reactions to catalyse the reversible hydrolysis of *S*-adenosylhomocysteine (SAH) to adenosine (Ado) and l-homocysteine (Hcy). In this study, SAHH protein was successfully cloned and purified with optimized, *Pichia pastoris* (*P*. *pastoris*) expression system. The biological activity results revealed that, among the tested compounds screened by ChemMapper and SciFinder Scholar, 4-(3-hydroxyprop-1-en-1-yl)-2-methoxyphenol (coniferyl alcohol, CAS: 458-35-5, ZINC: 12359045) exhibited the highest inhibition against rSAHH (IC_50_= 34 nM). Molecular docking studies showed that coniferyl alcohol was well docked into the active cavity of SAHH. And several H-bonds formed between them, which stabilized coniferyl alcohol in the active site of rSAHH with a proper conformation.

## Introduction

Serum homocysteine (Hcy) levels have been associated with age-related degenerative diseases, such as Alzheimer’s disease (AD), cardiovascular diseases, and stroke, etc.^1–3^ Elevated levels of Hcy can lead to elevated β–amyloid^4^, and increased levels of Hcy have been linked to morphological changes in hippocampal volume, to brain atrophy and to cognitive decline^5^. In the case of AD, high levels of Hcy appear approximately 5–8 years prior to the onset of Alzheimer’s dementia. Therefore, control of Hcy levels might present a novel strategy for AD prevention.

**Figure 1.**
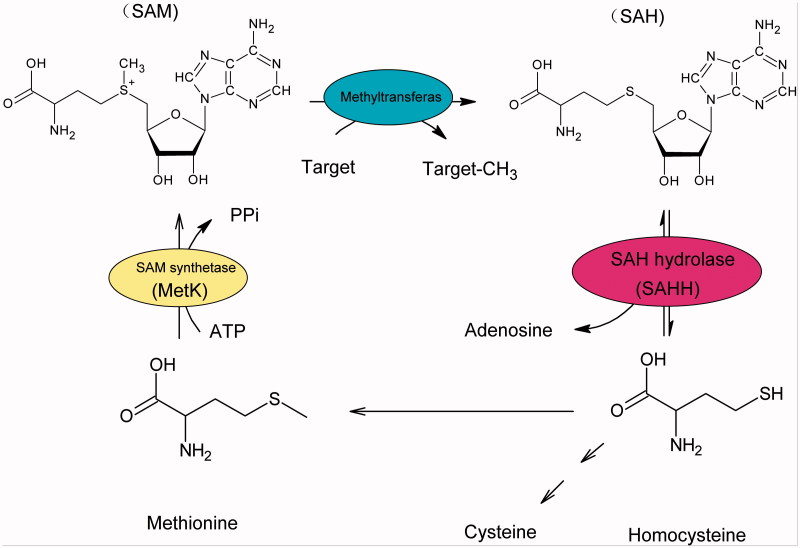
Activated methyl cycle (AMC) of mammalian cells.

In eukaryotes, Hcy mainly derives from hydrolysis of *S*-adenosylhomocysteine (SAH) to adenosine catalysed by *S*-adenosyl-homocysteine hydrolase (SAHH, E.C.3.3.1.1), as well as from diet^6^^,^^7^. Consequently, SAHH represents a potential drug target for the treatment of AD. Our initial objectives for this program were to characterize human *S*-adenosyl-homocysteine hydrolase *in vitro*, and to identify its efficient inhibitors. Furthermore, the inhibition of SAHH can lead to intracellular SAH accumulation, which triggers the negative feedback inhibition to suppress the *S*-adenosyl-l-methionine (SAM)-dependent transmethylation^8^ (Figure 1). Because of the prominent role of SAM-dependent transmethylation in capped methylated structure production at the 5′-terminus of viral mRNA, SAHH inhibitors could also be developed to be broad-spectrum antiviral agents^9–11^.

## Materials and methods

### Chemicals

Sepharose-4B, protein assay reagents, and chemicals for electrophoresis were purchased from Sigma–Aldrich (St. Louis, MO). Molecular compounds used for enzyme inhibition were purchased from J&K SCIENTIFIC Ltd. All other chemicals were of analytical grade and obtained from Sinopham Chemical Reagent Co., Ltd. (Jinan, China).

### Strains and culture medium

*Pichia pastoris* (*P. pastoris*) GS115 and vector pPIC9K were gifts from Dr Weifeng Liu’s laboratory of Shandong University. Plasmid pPIC9K was used to construct multi-copy expression vectors *in vitro*. All culture media, including minimal dextrose (MD), buffered minimal glycerol (BMGY) and buffered minimal methanol (BMMY) were prepared according to the *P*. *pastoris* expression manual (Invitrogen).

### Construction of recombinant pPIC9K-sahh

DNA manipulations were performed using standard methods^12^. *Pichia pastoris* was manipulated as described in the manual of the EasySelect^TM^*Pichia* Expression Kit (Invitrogen, Carlsbad, CA).

The optimized gene forward primer (5′AOX: GACTGGTTCCAATTGACAAGC) and reverse primer (3′AOX: GGCAAATGGCATTCTGACATCCT) were designed according to the predicted sequence of *sahh* (GenBank accession number BT006697.1). The optimized codon sequence of SAHH for *P*. *pastoris* was synthesized by GENEWIZ Inc. (Suzhou, China) and inserted into pPIC9K vector. The recombinant plasmid pPIC9K-*sahh* was verified by digesting with *EcoR* I and *Not* I, and then the target fragment was electrotransformed into *P*. *pastoris* obtained by linearizing the plasmid with *Bgl* II. To facilitate the upcoming purification of the recombinant *sahh*, a 6 × His tag-encoding sequence was in-frame fused to the 3′-end of the *sahh* coding sequence. Other than this, the nucleotide sequence for the restriction site *EcoR* I were sequentially incorporated at the 5′-end of the synthesized oligonucleotides and the *Not* I restriction site sequence were added to the 3′-end of the *sahh* coding sequence, respectively. The generation of the *P. pastoris* expression vector pPIC9K-*sahh* was verified by both restriction endonuclease analysis and direct nucleotide sequencing.

*Pichia pastoris* was transformed by electroporation^13^. In brief, 20 μL of *Bgl* II-linearized pPIC9K-*sahh* was mixed with 80 μL of competent *P*. *pastoris* cells. The cell mixture was kept on ice for 5 min, and then pulsed at 1500 V, 25 mF of capacitance and 200 U of resistance for 5 ms using a Gene Pulser Xcell apparatus (Bio-Rad Laboratories Inc., Philadelphia, PA). One milliliter of ice-cold sorbitol (1 M) was immediately added to the cuvette following electroporation. At last, each 50 μL of aliquots was spread on separate yeast MD plates containing 0.25 mg/mL of G418. Plates were incubated for 3–4 days at 30 °C.

The r*sahh*-positive *P*. *pastoris* transformants, which include *sahh* gene fragment and can grow on the medium containing G418, were screened by colony-PCR assay^14^. Single clone of G418-resistant *P. pastoris* transformants was selected and cultured on new yeast YPD. The culture supernatant was employed for PCR amplification using the pPIC9K vector-targeting primer pair. The PCR amplification was performed for 35 cycles at a condition of 94 °C for 60 s, 55 °C for 60 s and 72 °C for 90 s. Mock GS115 containing an empty pPIC9K and the recombinant plasmid pPIC9K-*sahh* were used as a negative and positive control, respectively. Then, the positive transformants were further cultured on new yeast YPDS plates containing 1.5 mg/mL of G418 to select high-copy expression strains.

### Expression and purification of S-adenosyl-homocysteine hydrolase

A single r*sahh*-positive *P*. *pastoris* colony was inoculated into 5 ml of BMGY medium (pH = 6.5) and grown at 29 °C in an agitating incubator at 200 rpm for 36 h. The cells were then transferred into 25 ml of BMGY medium to grow to reach an OD_600_ = 0.6–0.8. Cells were harvested by centrifugation at 14,000 × *g* for 15 min and resuspended in 25 ml of BMMY medium (50 ml system) to induce expression of rSAHH by adding pure methanol. Methanol was added every 24 h to reach a final concentration of 1.5% (*v/v*) during the 3–4 days induction period.

Affinity purification of rSAHH was performed using a Ni-NTA resin as previously described^15^ with minor modifications. Since rSAHH expressed by pPIC9K vector is expected to secret into the medium, the supernatant of culture (14,000 × *g*, 25 min) was loaded onto a Ni-NTA affinity resin column and equilibrated with binding buffer (0.05 M Tris-HCl, 10 mM imidazole at pH 8.0). Once the column was washed by using washing buffer (0.05 mM Tris-HCl, 100 mM imidazole at pH 8.0), the recombinant protein was eluted with elution buffer (0.05 M Tris-HCl, 200 mM imidazole at pH 8.0). The eluted protein was centrifuged to remove salt, and obtain the recombinant SAHH. Then, SDS-PAGE^16^ and coomassie brilliant blue were used for qualitative and quantitative determination of SAHH.

### SAHH activity determination

The activity of SAHH was expressed in units (U), which were defined as 1 μmol of SAH catalyzed by the enzyme per minute. Specific enzymatic activities (a_spec_) were calculated through the division of the enzymatic activities by the total protein amount used per assay and are expressed in U/mg. SAHH enzyme reacted with the SAH at different temperature and different pH, in order to identify its optimal reaction conditions. All of the values shown correspond to the mean values of at least three independent experiments, each of which was conducted in duplicate and all of the calculations were performed using Origin version 8.5 software and GraphPad Prism 5.

The enzyme kinetic parameters (*K_m_* and *K_cat_*) were calculated by least-squares fitting of the activity data at various substrate concentrations to the Michaelis–Menten equation with Origin version 8.5 software (Microcal Software, Northampton, MA).

### Inhibitors’ screening and molecular docking

Compounds structures were obtained through the ChemMapper platform (http://lilab.ecust.edu.cn/chemmapper/). Stock solution of compounds (1 mM) for enzyme inhibition was prepared in DMSO. Both enzymes and substrates are diluted in Tris-HCl (pH = 6.5) to specified concentration when used for IC_50_ determination.

Molecular docking was performed with autodock 4.2 program. Here, the X-ray crystallographic structure of SAHH (PDB code: 1A7A) was selected for docking analysis. The beta chain of protein and heteroatoms were removed from the original PDB file. The ligand coniferyl alcohol was docked into the active site of SAHH. During the docking process, the Lamarckian genetic algorithm (LGA) was applied to the conformational search for the protein–ligand binding structure. Among a series of docking parameters, the grid size was set to be 60 Å × 60 Å × 60 Å, and the grid space was the default value of 0.375 Å. The pose with the lowest free energy of binding was selected as the best binding mode. All the molecular graphic figures were generated by PyMOL software (http://www.pymol.org).

## Results and discussion

### Construction of recombinant vectors and screening of rsahh-positive transformants

**Figure 2.**
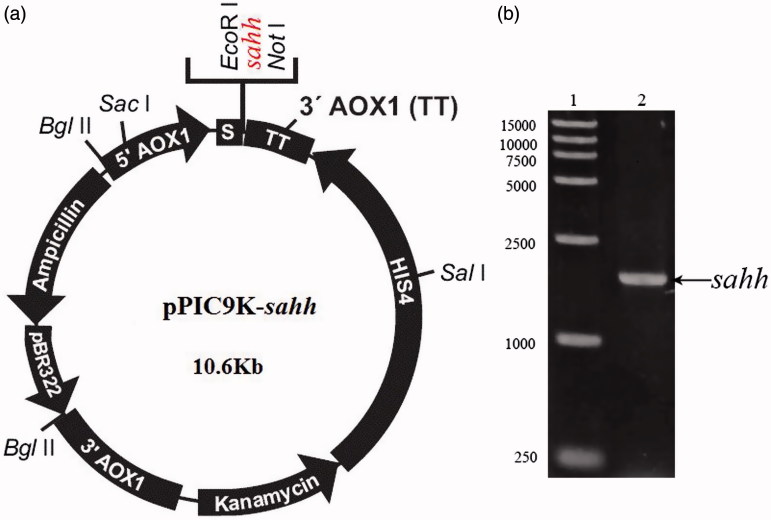
(a) Schematic diagram of the *P. pastoris* expression plasmid, pPIC9K-*sahh*. The nucleotide sequence encoding 6 × His-*sahh* was attached in-frame. (b) r*sahh*-Positive transformant selected by colony-PCR, 1. 15,000 bp DNA marker. 2. pPIC9K-*sahh*.

pPIC9K-*sahh* that contains a 1325 bp DNA fragment encoding a recombinant C-terminal 6 × His-tagged codon-optimized *sahh* was constructed in yeast (Figure 2(a)). Recombinants were verified with PCR using the primers. The size of the PCR amplified product was 1826 bp which is consistent with expected (Figure 2(b)). *Pichia pastoris* transformants were cultured on new yeast YPDS plates containing 0.5 mg/mL, 0.75 mg/mL, 1.0 mg/mL, 1.5 mg/mL of G418, respectively. Single colonies were picked out for PCR. Results showed that several single colonies grew well on medium with high concentration of G418, indicating that high-copy expression G418-resistant transformants were generated. *Pichia pastoris* has been used for the production of numerous recombinant proteins, and the strong AOX1 promoter that controls the target gene is tightly regulated and hence ideal for over expression^15^^,^^16^. And G418-resistant was chosen to obtain high-copy expression strains.

### Expression and purification of SAHH

**Figure 3.**
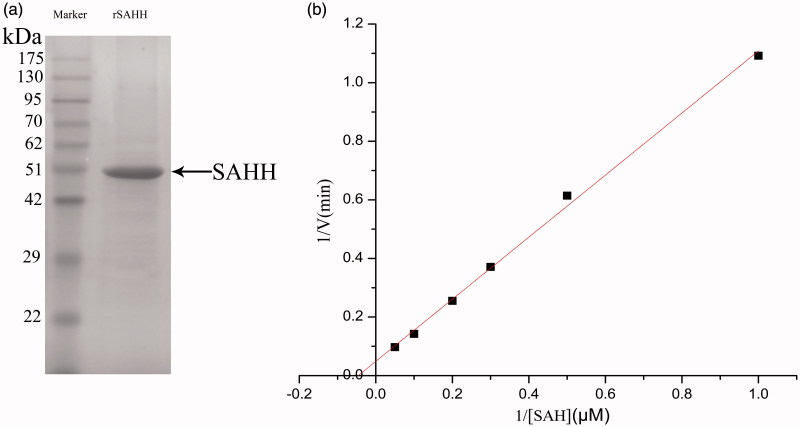
(a) Determining the molecular weight of the purified protein by SDS-PAGE. 1. Molecular weight protein standards. 2. A single band was detected with a molecular weight of approximately 47.5 kDa. (b) Michaelis–Menten equation of double-reciprocal. The red line shows linear relationship.

Based on the cultural condition of pH = 6.5, T = 22 °C, positive transformations were undergone the processes of fermentation, culture, induction, expression to obtain recombinant protein. After Ni-NTA affinity chromatography, the 6 × His-tagged rSAHH was purified by affinity chromatography, and affinity purification was determined by 10% SDS-PAGE. The size of secreted rSAHH is approximately 47.5 KDa, which is consistent with expected (Figure 3(a)). The concentration of rSAHH was determined to be 914.2 mg/L. Results demonstrated that the rSAHH peptide was successfully expressed.

### Determination of SAHH protein activity and parameters of enzyme kinetics

**Figure 4.**
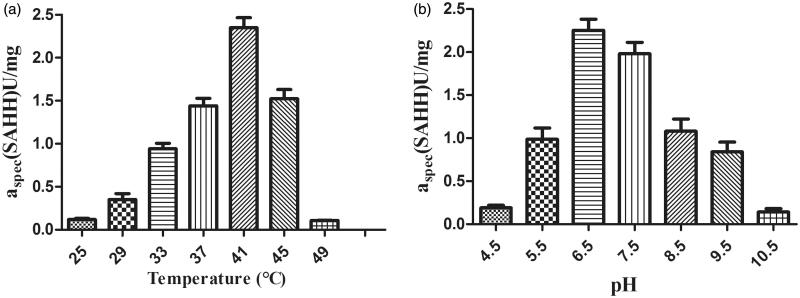
Effect of (a) temperature and (b) pH on SAH cleaving activity of purified recombinant full-length nucleosidase (rSAHH). Experiments were performed as described in the section “Materials and methods”. Each bar represents the average of three experimental determinations ± standard error.

SAH is hydrolysed by SAHH into Hcy and adenosine. And Hcy can be directly determined using Ellman reagent (DTNB)^17^. As shown in Figure 3(b), apparent *K_m_* values of the hydrolytic reaction were approximately 21.8 µM in SAHH, whereas the *V_max_* values were determined to be 22.9 µM/min. The enzyme kinetic parameters (*K_m_* and *K_cat_*) were calculated by least-squares fitting of the activity data at various substrate concentrations to the Michaelis–Menten equation with Origin version 8.5 software (Microcal Software, Northampton, MA). As shown in Figure 4(a,b), the optimum temperature for the SAHH enzyme was determined to be 41 °C, and optimum pH was found to be 6.5. The kinetic constants (*K_m_*, *V_max_*) for the SAHH-catalyzed hydrolysis of the substrate SAH were found to fit well into a Michaelis − Menten model (double-reciprocal form of the Michaelis–Menten equation $\text{1}/V=\left(K_{m}/V_{max}\right)•\left(\text{1}/\left[S\right]\right)+\text{1}/V_{max})$ with a high goodness of fit (*R*^2^= 0.99685).

### Design of SAHH inhibitors and biological screening

**Figure 5.**
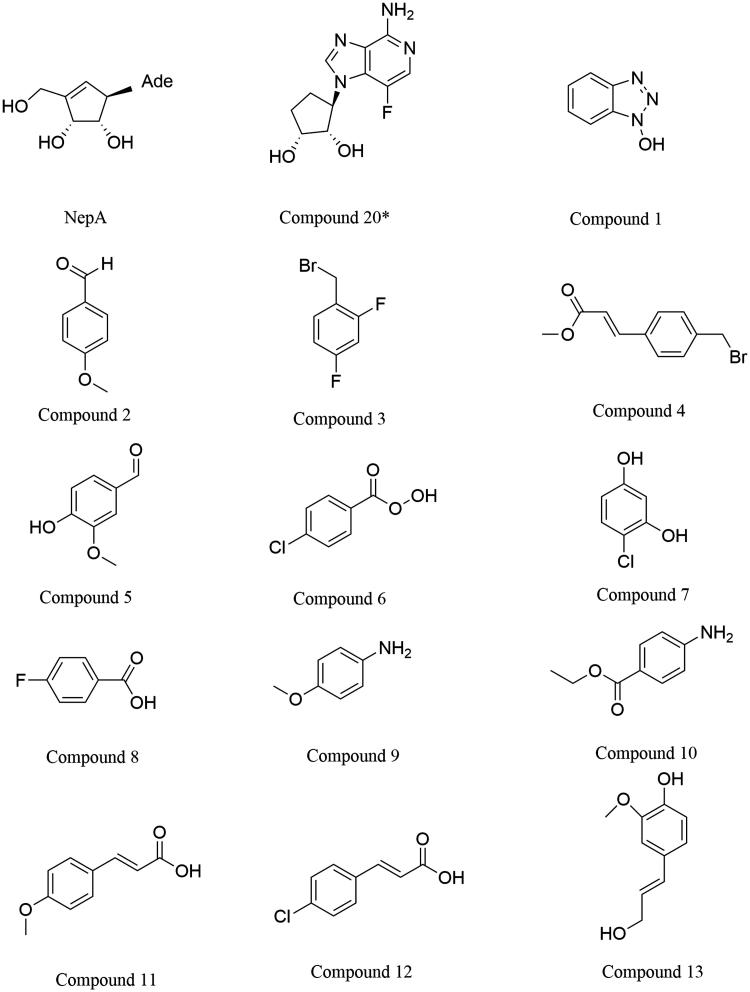
Structure of thirteen SAHH inhibitors.

ChemMapper is an online platform used to predict poly-pharmacology effect and mode of action for small molecules based on 3 D similarity computation. ChemMapper contains >350,000 chemical structures with bioactivities and associated target annotations (as well as  >3,000,000 non-annotated compounds for virtual screening)^18^. Picking up the user-provided chemical structure as the query, the topmost similar compounds in terms of 3 D similarity are returned with associated pharmacology annotations. Neplanocin A (NepA) is a natural product known to inhibit SAHH with nanomolar activity^19^ (Table 1). However, NepA is cytotoxic upon long term exposure and shows adenosine deaminase and adenosine kinase activity. Compound 20*(CAS:1214735-71-3)^19^, a novel inhibitor for SAHH, was generated by Converso. Converso reported Compound 20* was in a position to reduce brain homocysteine in a dose-dependent manner. Inspired by Compound 20*, we obtained 338 different compounds through the ChemMapper platform (http://lilab.ecust.edu.cn/chemmapper/). Eventually, 13 compounds (Figure 5, 1–13) were singled out by the ZINC and SciFinder from the 338 selected compounds. The inhibitory effects of the thirteen small organic compounds were tested (IC_50_ are given in Table 1). We found that the SAH had a reduced hydrolysis rate with increasing inhibitor concentration and that the IC_50_ values of the tested inhibitors were comparable to those previously reported. Among them, compound 13, which is determined to be coniferyl alcohol (4–(3-hydroxyprop-1-en-1-yl)-2-methoxyphenol, CAS: 458–35-5, ZINC: 12359045), has displayed considerable inhibitory effects (IC_50_ = 34 nM). Actually, coniferyl alcohol, as a component of therapeutic agents, is being tested as a drug in treatment for breast cancer and prostate cancer^20^. Such results have been encouraging and suggest that coniferyl alcohol might be a valuable molecular tool for further interrogation of this promising pathway for the treatment of age-related degenerative diseases.

**Figure 6.**
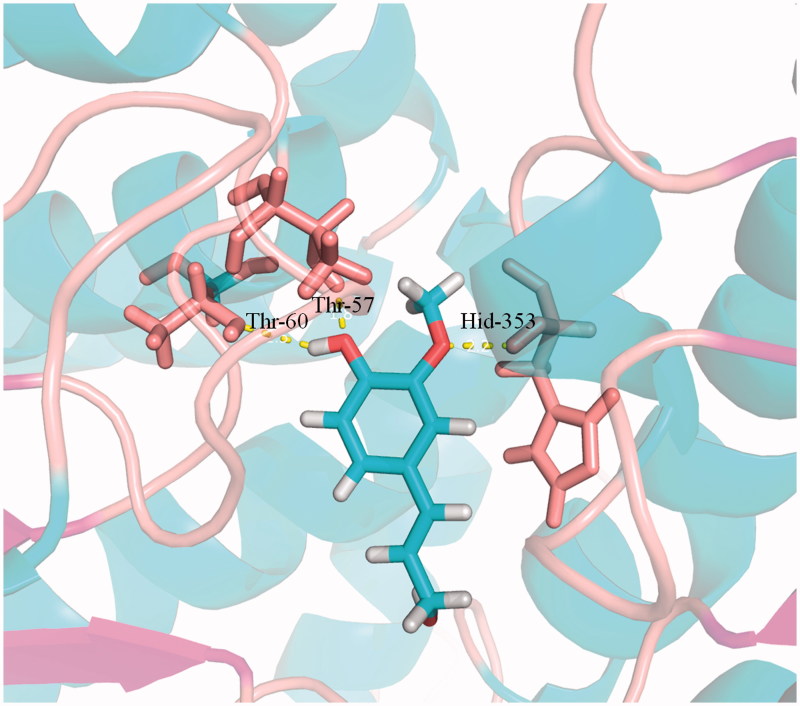
Docking simulations showed the detail of coniferyl alcohol binding site in SAHH active pocket. Hydrogen bonds are represented by dotted pink lines.

**Table 1.** Calculated IC_50_ values of the most potent compounds determined for SAHH and compared with NepA and Compound 20*.

Compound	SAHH IC_50_ (nM)
NepA	1.5*
20*	40*
1	1245
2	2131
3	913
4	814
5	1103
6	565
7	1110
8	679
9	899
10	1399
11	986
12	571
13	34

* Data were from literature 19.

To test the feasibility of our design, we further performed a molecular modelling study to explore the mode of molecular interaction of coniferyl alcohol at the active site. As shown in Figure 6, coniferyl alcohol can be well docked into the active cavity of SAHH (PDB code: 1A7A). Several H-bonds obviously formed between coniferyl alcohol and the SAHH. The C1′ OH in the benzene ring could form an H-bond with Thr-60 and Thr-57. In addition, C2′ methoxy formed an H-bond with Hid353. These H-bonds stabilized coniferyl alcohol in the active site with a proper conformation. The predicted binding energy for ligand coniferyl alcohol was −10.18 kcal mol^−1^. Both theoretical analysis and experimental results showed that coniferyl alcohol could be well bound to SAHH and that coniferyl alcohol is likely to be selected as the inhibitor for SAHH.

SAHH, along with SAHN (*S*-adenosylhomocysteine nucleosidase, EC 3.2.2.9), which is involved in the recycling pathway of adenine, sulphur, and methionine in pathogens and produces a universal quorum sensing signal, autoinducer-2 (AI-2), have become attractive targets for drug design. Inhibition of SAHN would be expected to remove the quorum sensing autoinducer molecules, thus reducing the drug-resistance of pathogens^21^^,^^22^. SAHH plays a critical role in the mammalian methylation process, which makes it a potential drug target in the discovery of antiviral agents and in the treatment of age-related degenerative diseases. There is likewise increasing interest in determining its activity in the biological and clinical fields with chemosensors but this has had limited success so far^7^. Here we report that recombinant human SAHH has been successfully expressed in *P. pastoris*, and its biochemical characteristics are analyzed in detail. Furthermore, a potential SAHH inhibitor of coniferyl alcohol, has been singled out from 338 possible compounds, and displays efficient inhibitory effects to human SAHH enzyme activity *in vitro* (IC_50_ = 34 nM). Additionally, coniferyl alcohol has also shown to have better binding affinity with human SAHH protein in computational docking studies, which verifies its potential role for further interrogation in the treatment of age-related degenerative diseases.
